# Improving experiences of neglected tropical diseases of the skin: Mixed methods formative research for development of a complex intervention in Atwima Mponua District, Ghana

**DOI:** 10.1371/journal.pgph.0002833

**Published:** 2024-06-13

**Authors:** Daniel Okyere, Edmond Kwaku Ocloo, Lucy Owusu, Yaw Ampem Amoako, Ruth Dede Tuwor, Eric Koka, Jacob Novignon, Adwoa Asante-Poku, Ishaque Mintah Siam, Emmanuel Kyei Afreh, Abigail Agbanyo, Richard Adjei Akuffo, Solomon Gyabaah, Michael Ntiamoah Oppong, Katherine E. Halliday, Hope Simpson, Joseph Timothy, Michael Marks, Maria Zuurmond, Stephen L. Walker, Rachel L. Pullan, Collins Stephen Ahorlu, Richard Odame Phillips, Dorothy Yeboah-Manu, Catherine Pitt, Jennifer Palmer, SHARP collaboration

**Affiliations:** 1 Noguchi Memorial Institute of Medical Research, University of Ghana, Accra, Ghana; 2 Department of Sociology and Anthropology, University of Cape Coast, Cape Coast, Ghana; 3 Kumasi Centre for Collaborative Research, Kwame Nkrumah University of Science & Technology, Kumasi, Ghana; 4 School of Medicine and Dentistry, Kwame Nkrumah University of Science & Technology, Kumasi, Ghana; 5 Department of Economics, Kwame Nkrumah University of Science & Technology, Kumasi, Ghana; 6 Faculty of Infectious and Tropical Diseases, London School of Hygiene & Tropical Medicine, London, United Kingdom; 7 Hospital for Tropical Diseases, University College London Hospital, London, United Kingdom; 8 Division of Infection and Immunity, University College London, London, United Kingdom; 9 Faculty of Epidemiology and Population Health, London School of Hygiene & Tropical Medicine, London, United Kingdom; 10 Faculty of Public Health and Policy, London School of Hygiene & Tropical Medicine, London, United Kingdom; University of Washington Seattle Campus: University of Washington, UNITED STATES

## Abstract

Integrated approaches to managing co-endemic neglected tropical diseases (NTDs) of the skin within primary healthcare services are complex and require tailoring to local contexts. We describe formative research in Atwima Mponua District in Ghana’s Ashanti Region designed to inform the development of a sustainable intervention to improve access to skin NTD care. We employed a convergent, parallel, mixed-methods design, collecting data from February 2021 to February 2022. We quantitatively assessed service readiness using a standardised checklist and reviewed outpatient department registers and condition-specific case records in all government health facilities in the district. Alongside a review of policy documents, we conducted 49 interviews and 7 focus group discussions with purposively selected affected persons, caregivers, community members, health workers, and policy-makers to understand skin NTD care-seeking practices and the policy landscape. Outside the district hospital, skin NTD reporting rates in the surveyed facilities were low; supply chains for skin NTD diagnostics, consumables, and medicines had gaps; and health worker knowledge of skin NTDs was limited. Affected people described fragmented care, provided mostly by hospitals (often outside the district) or traditional healers, resulting in challenges obtaining timely diagnosis and treatment and high care-seeking costs. Affected people experienced stigma, although the extent to which stigma influenced care-seeking behaviour was unclear. National actors were more optimistic than district-level actors about local resource availability for skin NTD care and were sceptical of including traditional healers in interventions. Our findings indicate that improvement of the care cascade for affected individuals to reduce the clinical, economic, and psychosocial impact of skin NTDs is likely to require a complementary set of interventions. These findings have informed the design of a strategy to support high-quality, integrated, decentralised care for skin NTDs in Atwima Mponua, which will be assessed through a multidisciplinary evaluation.

## Introduction

Several Neglected Tropical Diseases (NTDs) manifest primarily in the skin and are referred to as ‘skin NTDs’ [1]. In Ghana, many skin NTDs (including Buruli ulcer [BU], leprosy, yaws, and scabies) principally affect children and adolescents, especially in poor and marginalized communities with limited access to health services, water, and sanitary living conditions [2]. Access to accurate, early diagnosis and appropriate treatment is crucial to minimize long-term consequences, which may include scarring [3]; loss of function affecting limbs, digits, and joints [3]; complications affecting bones and nerves [4]; impaired mental health [5]; and economic hardship [6].

The World Health Organization’s (WHO) 2021–2030 NTD Roadmap promotes integrated approaches to diagnose and manage co-endemic skin NTDs [7]. In 2021, the Ghana Health Service (GHS) updated its NTD Masterplan, promoting integration of disease-specific, centralised programmes within primary healthcare. The operational framework includes training and equipping health workers in diagnosis, treatment, and management of cases and contacts in peripheral health facilities, communities, and schools [8]. Integrated approaches are complex and require tailoring to local contexts, including disease distributions, existing healthcare provision, and the ways individuals and communities experience diseases and care services.

Many factors can influence whether or not affected people receive appropriate care for skin NTDs, but existing literature is sparse, often draws conclusions from a single research discipline or methodological approach and tends to focus on individual diseases. Studies of household experiences across multiple skin NTDs have been conducted focusing on children in Côte d’Ivoire [9] and hospital care in Liberia [10], but not in Ghana, where single-disease studies [11], primarily of BU [12–14], predominate. Even for single diseases, illness representations and how they affect healthcare decision-making are known to vary widely across cultures and contexts [13]. Several studies have evaluated activities to “find” cases of skin NTDs in Ghana and elsewhere [15–20], but evidence is limited on the care cascade challenges and preparedness of routine primary health services to provide integrated care for multiple skin NTDs [21]. For GHS to implement sustainable, decentralised, integrated skin NTD care, improved understanding is needed of the potential of the health service to reach affected people early and provide appropriate care within specific local contexts.

We therefore conducted formative research to inform development of a sustainable strategy in a pilot district in Ashanti Region, as part of a three-phase intervention study. In this formative stage, we investigated the local health system’s readiness to provide care; perceptions and experiences of skin NTDs and services by people affected, their families, and the wider community; and perspectives on policy and practice challenges of key actors at district, regional, and national levels. Work was conducted in consultation with GHS actors, and led by the Skin Health Africa Research Programme (SHARP).

## Methods

### Study setting

Atwima Mponua District (population 155,254) [22] was selected for the study because anecdotal evidence provided by district hospitals and regional referral facilities in neighbouring districts suggested the presence of both BU and yaws and possibly leprosy; no GHS interventions specifically targeting skin NTDs had been conducted; and, to our knowledge, the district had not received previous investment in skin NTDs from non-government organizations, private donors or other development partners.

**Fig 1:**
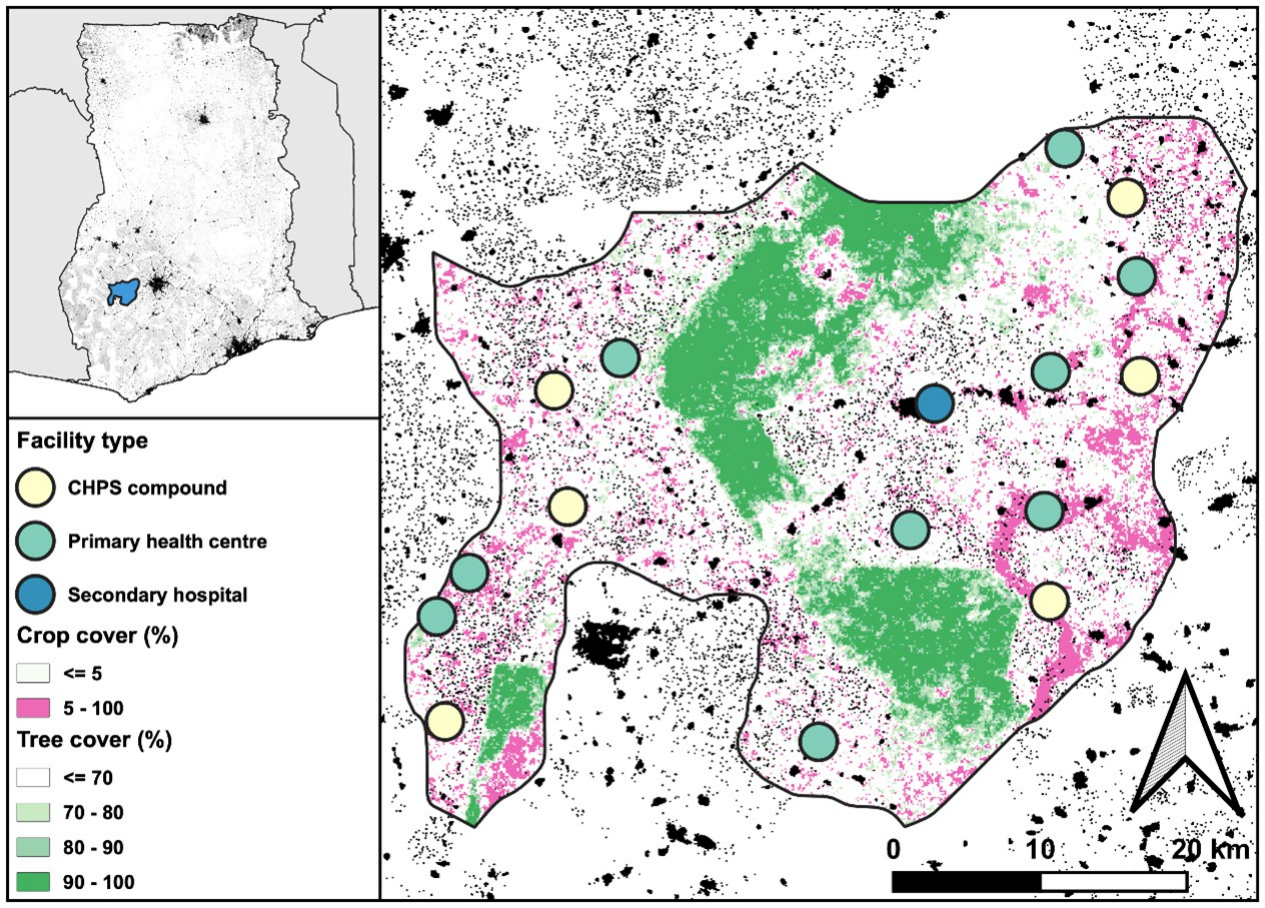
Map of Atwima Mponua District showing the location of GHS facilities by type. Background shading reflects population density (in black) alongside tree (green) and crop (pink) cover based on satellite imagery. The inset map shows the location of Atwima Mponua in Ghana. Basemap was generated in qGIS using district boundary files provided by GADM (https://gadm.org/) and landcover and population data provided by ESA WorldCover project (modified Copernicus Sentinel data (2021) processed by ESA WorldCover consortium: https://worldcover2021.esa.int/download) and GRID3 (Ghana Settlement Extents v2.0: https://data.grid3.org/datasets/GRID3::grid3-gha-settlement-extents-v2-0/explore). These data sources are all provided free of charge, without restriction of use (CC BY 4.0). For information on licensing: https://gadm.org/license.html, https://worldcover2021.esa.int/download, https://data.grid3.org/datasets/GRID3::grid3-gha-settlement-extents-v2-0/about.

Atwima Mponua is the westernmost district of Ashanti Region. The district capital, Nyinahin, lies 64km from the regional capital, Kumasi, and is on the main Bibiani-Kumasi highway. Roads through the rest of the district are mostly unpaved and can become impassable in the rainy season. Four forest reserves cover 40% of the total land area. The population is predominantly engaged in small-scale agriculture (crop farming, fishery, and forestry) with some informal alluvial mining. The main language spoken is Asante-Twi. The district has a public hospital in Nyinahin and 15 peripheral GHS facilities: 9 health centres and 6 community-based health planning and services (CHPS) facilities (Fig 1). In neighbouring districts, Nkawie Hospital runs a limited BU clinic, and the Komfo Anokye Teaching Hospital in Kumasi provides more comprehensive skin NTD referral services. Some people also obtain care from traditional healers.

### Study design

**Fig 2:**
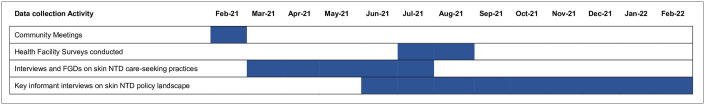
Timeline of data collection activities.

We employed a convergent, parallel, mixed-methods design using a pragmatic approach to generate a deeper and more holistic understanding of the multifaceted research question. We collected data from February 2021 to February 2022, as described further below (Fig 2, Table 1). Prior to our main data collection activities, we held community meetings in February 2021 to introduce the study. In July and August 2021, we conducted a quantitative cross-sectional study of health service provision comprised of a service availability and readiness assessment survey [23, 24] and review of routine health facility records. In parallel, from March 2021 to February 2022, we conducted in-depth interviews (IDIs) and focus group discussions (FGDs) with purposively selected target groups to describe common skin NTD care-seeking practices in the district (conducted March-July 2021) and intervention challenges in the policy landscape (conducted July 2021—February 2022). The latter was complemented by a review of policy documents. Detailed qualitative methods are reported using the COnsolidated criteria for REporting Qualitative research (COREQ) Checklist (S2 Checklist) and summarised below. Policy makers from the GHS helped to ensure the study design and questions were situated within the current policy context and facilitated approvals and introductions in the district.

**Table 1:** Summary of data collected.

Data collection tool	Number	Sampling strategy
**Community meetings**	14 meetings	Purposive
**Service availability and readiness assessment**	16 health facilities	Exhaustive (all public facilities in the district)
**Health facility records review**	9 health centres + 1 hospital	Exhaustive (all health centres + hospital in the district)
**In-depth interviews**	33 health workers	Purposive (2 per facility + district disease control officer)
	8 affected individuals	Purposive (via snowballing beginning with health workers)
	6 household caregivers	Purposive (via snowballing beginning with health workers)
	3 community members involved in care	Purposive (via snowballing beginning with health workers)
**Focus group discussions**	7 discussions	Purposive (via interviews and snowballing beginning with disease control officer)
**Key informant interviews**	8 regional and national actors	Purposive (based on professional roles)
**Policy document review**	6 key national and international documents	Purposive (based on relevance to skin NTDs)

### Data collection

#### Stakeholder and community engagement

We began with a series of 14 meetings with community representatives (local leaders and health facility leads) across Atwima Mponua. Held in Asante-Twi and recorded using field notes, the meetings introduced and sought permission for the study, collected views on the relative importance of skin problems (such as Buruli ulcer and leprosy), proposed a study design, and discussed the value of future intervention plans.

#### Health service readiness survey and records review

Structured service availability and readiness assessment surveys were conducted for general services based on standardised WHO indicators at all 16 GHS health facilities; the health facility was the unit of assessment [23, 24]. We additionally developed specific question modules for skin NTDs of interest, which assessed the availability of trained clinical staff as well as diagnostics, treatment, and case management services for BU, leprosy, and yaws. Each domain was defined based on the presence or absence of the recommended standard of care as defined by the Ministry of Health, such as the availability of staff trained in diagnosis and management for each condition, and valid serological rapid diagnostic tests for yaws. During each facility assessment, study staff surveyed the individuals in charge of outpatient care, or an alternative, suitably informed staff member such as the facility in-charge; in most cases, one individual completed the entire survey.

We reviewed routine health surveillance records at the district hospital and all health centres for a 24-month period 1 Jan 2019 to 31 Dec 2019 (pre-Covid-19) and 1 August 2020 to 31 July 2021. CHPS compounds were excluded because of the observed low use of health data reporting systems. Health facility staff were initially consulted to identify all methods in use at the facility for recording data for BU, leprosy, yaws and common skin problems. We then reviewed all disease-specific case reporting forms, where available, and extracted case-level data (including demographics, clinical staging, laboratory confirmation, and number of times seen, when available). Additionally, general outpatient department registers and aggregated reports (i.e. monthly and annual totals) were reviewed, with all common skin disease and skin NTD cases tallied. All health service data were collected using Open Data Kit [25], encrypted, and stored on password-protected servers.

#### Skin NTD care-seeking practices

Ghanaian members of the research team iteratively collected qualitative data on care-seeking in English or Asante-Twi, according to the participant’s preference (S2 Checklist). As limited information was available through official case reporting to the district, we first conducted exploratory interviews with one health worker at each of the 9 health centres and hospital to help identify individuals with presumed or confirmed skin NTDs, who were invited to participate. Snowball sampling was used to identify additional affected individuals and their caregivers.

In-depth interviews with health workers (33 in total, including the 9 described above) explored their strategies to detect cases in the community and health facilities, manage patients and ensure treatment adherence, and their understanding of stigma. We explored their hypotheses about patient experiences to inform further research with affected individuals and their families. The 33 health workers comprised two personnel from each of the 16 health facilities in the study area and the district disease control officer. For these interviews, we sought participants who were actively involved with care for persons affected by skin NTDs through recommendation from each health facility lead.

Interviews with 8 affected individuals explored their lived experiences of skin NTDs (7 confirmed or presumed to be affected by BU, 1 confirmed to have leprosy), including decision-making and beliefs affecting care-seeking, economic burdens on households, and experiences of stigma. Interviews were also conducted with six household caregivers and three other community members involved in these individuals’ care (a traditional healer, teacher, and community volunteer). These latter informants could speak more broadly about locations where diagnosis and care for skin NTDs happen outside health facilities and referral linkages between them. They also introduced people they knew or presumed to be affected by a skin NTD, contributing to the snowball sampling.

Seven FGDs were conducted with health workers (2 groups, some of whom had participated in IDIs), affected people (1 group, all with BU, who also gave IDIs), and community members (4 groups from catchment areas of facilities reporting cases) to explore community-level awareness of and attitudes towards skin NTDs and common skin conditions and perspectives on local care, including barriers to care-seeking. For these discussions, health workers were recruited from the two facilities with the highest recorded number of skin NTD cases in the previous 12 months and were selected based on recommendations from the district disease control officer.

#### Skin NTD policy landscape

Eight key informant interviews were held in English with regional and national-level actors (disease control programme managers, health officials from other relevant departments, and representatives of non-governmental skin NTD programmes in other districts) to understand their perspectives on skin NTD policy implementation, existing gaps and challenges in the provision of care, potential intervention priorities, and opportunities for improvement. Key policy documents relevant to skin NTDs and service decentralisation referred to by our informants [4, 7, 8, 26–28] were analysed and the informant’s opinion on how these documents have shaped the policy landscape recorded.

### Data analysis

#### Quantitative data analysis

Health facility data from the service availability and readiness assessment survey were summarised using RStudio (version 4.0.4). The main outcomes were coded as binary indicators describing characteristics of health facilities and service provision for basic health services (including human resources, capital infrastructure and availability of essential consumables) and skin NTD services (including availability of guidelines and supplies, management and training systems, and facility readiness to diagnose and treat conditions). For the records review, we tabulated the cases reported across the case reporting forms and OPD registers. Results were summarised by facility and for the district with frequencies and percentages. In cases where there were discrepancies between recording types, the greater case load was used.

#### Qualitative data analysis

Qualitative data were transcribed in English (with translation by the study team as necessary) coded thematically, and managed with MAXQDA software. A coding framework was developed collaboratively and included the following pre-agreed themes: transmission discourses, experiences of care-seeking and provision (including ways of recognising skin NTDs and interpreting diagnoses), economic burdens (financial and opportunity costs of care-seeking, other costs to households, and coping strategies), experiences of stigma (internalised/‘felt’ and social/‘enacted’ stigma), its structural manifestations, and coping strategies) and opinions on intervention possibilities. Policy documents were thematically analysed alongside transcripts from interviews. We considered them as written objects created by negotiation, reflecting the different experiences, aims, and interests of contributing stakeholders, but which do not tell the whole story [29]. Data were first coded to allocate material across themes and then distributed to a thematic lead researcher who was responsible for further coding, synthesis, and interpretation through discussion with the wider team. In addition, from sufficiently detailed interviews about the care-seeking pathways of people with BU, we identified key events, locations and people involved in their care and present them graphically.

#### Synthesis and interpretation

Findings were discussed at a meeting in March 2022 to identify commonalities and points of divergence in themes arising from different study phases, methods, and theoretical orientations. Key national and district-level stakeholders were also invited to this meeting to reflect on findings and future intervention possibilities, which are described separately [30]. We present findings organized into four themes: the health service context, perceptions and experiences of skin NTDs, care-seeking pathways, and the policy landscape.

### Ethical approval and consent

This study was approved by the institutional review boards of the Noguchi Memorial Institute for Medical Research, Ghana (CPN: 022/20-21 and CPN: 022/20-21 revd. 21) and the London School of Hygiene and Tropical Medicine, UK (22604). Individuals who participated in readiness surveys, IDIs and/or FGDs provided written informed consent to participate and for inclusion of their contributions in subsequent publications. Participants in meetings gave permission for notes to be taken and were encouraged to identify any information that should not be used.

#### Inclusivity in global research

Additional information regarding the ethical, cultural, and scientific considerations specific to inclusivity in global research is included in the Supporting Information (S1 Checklist).

## Results

### Health service context

#### Basic health service readiness

The district health facility density was 1 facility per 10,000 population, half the WHO recommended minimum. The number of clinically trained health workers (18.9 per 10,000 population) was 78% of the WHO-recommended minimum [24]. Only the district hospital employed physicians (Table 2). Fifteen of 16 facilities had an improved water source and electricity. Only the district hospital, two health centres, and one CHPS compound had a computer, which affected data reporting formats. Overall, routine diagnostic capacity in health facilities for diseases other than skin NTDs was good; for example, 14 of 16 had HIV and syphilis rapid diagnostic tests. Essential medicines such as oral antibacterial drugs were stocked by all facilities, but only one of six CHPS compounds had a first aid kit.

**Table 2:** Service availability and readiness assessment summary.

Category	Resource	District hospital (N = 1)	Health centres (N = 9)	CHPS (N = 6)
**Basic health services**				
Human resources	Physicians total across facility type)	**2**	**1**	**0**
	Core workforce ^1^ (median per facility [range])	**117**	**11 [4–32]**	**4.5 [3–8]**
Capital infrastructure	Improved water source	1	9	5
	Electricity	1	9	5
	Computer	1	3	1
	Private consultation room	1	8	5
Availability of tracer consumables	HIV rapid diagnostic tests in stock	1	8	5
	Syphilis rapid diagnostic tests in stock	1	8	5
	Oral antibacterial drugs in stock	1	9	6
	First aid kit in stock	1	7	1
**Skin NTD services**				
Training	BU: ≥1 staff member trained in treatment and/or diagnosis in past 2 years	0	2	3
	Yaws: ≥1 staff member trained in treatment and/or diagnosis in past 2 years	1	2	0
	Leprosy: ≥1 staff member trained in treatment and/or diagnosis in past 2 years	1	1	3
Staff knowledge of key signs and symptoms	BU: recognises all clinical forms (nodule, plaque, oedema, osteomyelitis and ulcer)	0	2	0
	Yaws: recognises both major clinical forms (papilloma and ulcer)	1	1	0
	Leprosy: recognises 3 cardinal signs^2^	1	0	0
Diagnostic testing or confirmation	BU: Swab or FNA samples collected for confirmatory testing outside the district	1	2	0
	Yaws: At least one yaws laboratory diagnostic 3 is offered at facility	1	3	0
	Leprosy: Diagnosis via syndromic cardinal signs offered at facility	1	2	0
Treatment and care	BU: Rifampicin + Clarithromycin 56-day course **offered** at facility	1	4	2
	BU: Rifampicin + Clarithromycin 56-day course **in stock** at facility	0	0	0
	Yaws: Azithromycin or benzathine penicillin treatment **offered** at facility	1	5	1
	Yaws: Azithromycin or benzathine penicillin treatment **in stock** at facility	1	2	0
	Leprosy: Leprosy multidrug treatment **offered** at facility	1	0	0
	Leprosy: Leprosy multidrug treatment **in stock** at facility	0	0	0
	Wound care materials (including dressings, analgesics & antiseptics) **in stock**	0	4	1

^1^ Core workforce includes physicians, non-physician clinicians, registered nurses, and midwives–but excludes community health workers, case managers and community-based surveillance volunteers.

^2^ Cardinal signs of leprosy include: loss of sensation in a pale or reddish skin patch; enlarged peripheral nerve, with loss of sensation and/or weakness of muscles supplied by that nerve; microscopic detection of bacilli in a slit-skin smear.

^3^ Options include Treponemal Antibody Test, Rapid Plasma Reagin Test, Rapid Syphilis Test or DPP Point of Care Test.

FNA: Fine needle aspirate.

#### Health service readiness to provide care for skin NTDs

The district hospital was the only facility providing diagnostic and treatment services for endemic skin NTDs, but BU services were not comprehensive. BU samples were analysed at Kumasi Hospital, and patients were expected to attend other health facilities for dressings. Physical rehabilitation services were not available and severe wounds requiring skin grafts were referred outside the district. Few of the 9 health centres provided diagnostic services for BU (n = 2), leprosy (n = 2), and yaws (n = 3). Syphilis rapid tests, stocked in most facilities, can be used to aid diagnosis of yaws but it was reported they were not used for this purpose. The availability of anti-microbial therapies for skin NTDs was limited. For BU, 4 health centres reported they could dispense drugs, but none kept these in stock. Azithromycin for yaws was in stock in 2 of 5 health centres that reported they could dispense it. Leprosy multi-drug therapy was only available at the hospital and was not kept in stock. Only three health centres stocked BU wound care essentials: dressings, antiseptics, and analgesics. A fourth facility provided wound dressing services, but dressings were out of stock. CHPS facilities are not expected to deliver skin NTD services; 1 of 6 stocked wound care materials.

Health worker training on skin NTD diagnosis and management in the previous two years was low. Health workers had received training on BU at 5 facilities and for yaws at 3 facilities. No health workers had received training on leprosy. Knowledge of signs of skin NTDs appeared low at facilities other than the hospital.

#### Health worker practices to manage skin NTDs

Many health workers reported not having received formal training in skin problems after their initial clinical education. They had little confidence in their ability to make clinical diagnoses and this, combined with lack of diagnostic tests, meant patients needing skin care tended to be referred to hospitals outside the district:

*“We cannot diagnose whether this is Buruli ulcer or yaws. We call it nodules; it looks like boils. We do the little we can for him or her and then refer them to the Nkawie Government Hospital to check.”*

*[065 FGD, Heath Worker at a CHPS facility]*

Several health workers felt their lack of involvement in skin NTD diagnosis contributed to people preferring alternative care, diagnostic delays, unnecessary complications, and underestimates of the district disease burden.

Health workers who attempted to make provisional skin NTD diagnoses described using self-directed learning practices. Sometimes these practices built on experiential learning by discussing cases with peers or with district disease control officers during their visits. Others learned from educational resources they came upon by chance, such as leprosy campaigns and documentaries by NGOs working in other districts. Online images of skin NTDs were then used to ‘refresh’ learning to facilitate diagnosis during consultations.

#### Health service data on skin NTD prevalence

Over the 24-month period, the district recorded 8 confirmed BU cases (new case detection rate: 0.25 per 10,000 people per year), 1 confirmed leprosy case (0.032 per 10,000 per year) and 30 reports of potential yaws cases (0.9 per 10,000 per year, all diagnosed using syndromic but not serological criteria). Nearly all were reported by the district hospital (7 BU [88%], 1 leprosy [100%] and 27 yaws cases [90%]). Most yaws cases were identified following an outbreak investigation in one village; the district disease control officer initiated the investigation after a mother sought care for her child with yaws at the district hospital. Seven (of 9) health centres did not record any confirmed or presumed BU, 6 did not report any yaws, and 8 did not report any leprosy in the preceding 2 years.

Community representatives described BU, leprosy, and yaws as silent, neglected, or “hidden” problems. Cases known to the community were often unknown to the health service, which was attributed, in part, to a lack of visible services.

### Perceptions and experiences of skin NTDs

#### Knowledge and causation beliefs within communities

Collective signs and symptoms of leprosy (*kwata*), yaws (*dei* or *gyatoo*) and scabies (*dweeba dweeba*, from “scratching”) were identified by specific words in Twi, while Buruli ulcer was simply known as *Buruli*. Children were said to be most often affected by skin problems through greater exposure to dirty water and other infected children and because of weaker immune systems. Adults were said to be at risk of skin problems through injuries sustained during farming, exposure to chemicals during mining (men and women), and use of skin-lightening creams (mainly women).

Moral associations with skin NTDs were complex, depending on disease-specific causation narratives, the type of person affected, and how they might attract diseases as a supernatural problem. BU was commonly conceptualised as a complication stemming from “*little scratches*” or chemical-based injuries that are hard to avoid, so was generally not deemed the fault of affected people. Leprosy was associated with older age, or with older women suspected of being witches, while yaws was said to affect unhygienic families or children who lack proper “*parental care*” [Int 46, health worker].

Some skin conditions, like many forms of ill health in Ghana, may also be attributed to punishment (bad karma) for past misdeeds (*abonsam yare*, literally ‘Devil/Satan’s curse/illness’) or to witchcraft involving disease-causing curses paid for by vindictive people (*ntɔ yareɛ*, literally ‘bought/contract curse/illness’). These conditions were said to be a curse to punish individuals for taboo behaviour, retribution for a family conflict, or because of jealousy including of young people expected to be leaders or successful in the future.

Knowledge of the features of skin NTDs varied widely. Knowledge about yaws appeared restricted to communities with a recent history of cases. Knowledge of leprosy was concentrated in older people who had seen affected people in the past. In contrast, discussions of BU often involved detailed descriptions of how to recognise its early signs and expected progression during traditional and biomedical interventions. For example, a women’s group described how BU can appear differently on the outside compared to the inside once it is cut into:

*“It comes on the skin like a normal ‘ntatia hyire’ [rash]. It is round on the skin, and […] it would just be there and be expanding underneath if you don’t take it to the hospital early for treatments. The day you’ll go, you’ll see that it has destroyed the tissues inside of your leg but outside it looks fine.”*

[Participant 8, women’s FGD]

**Fig 3:**
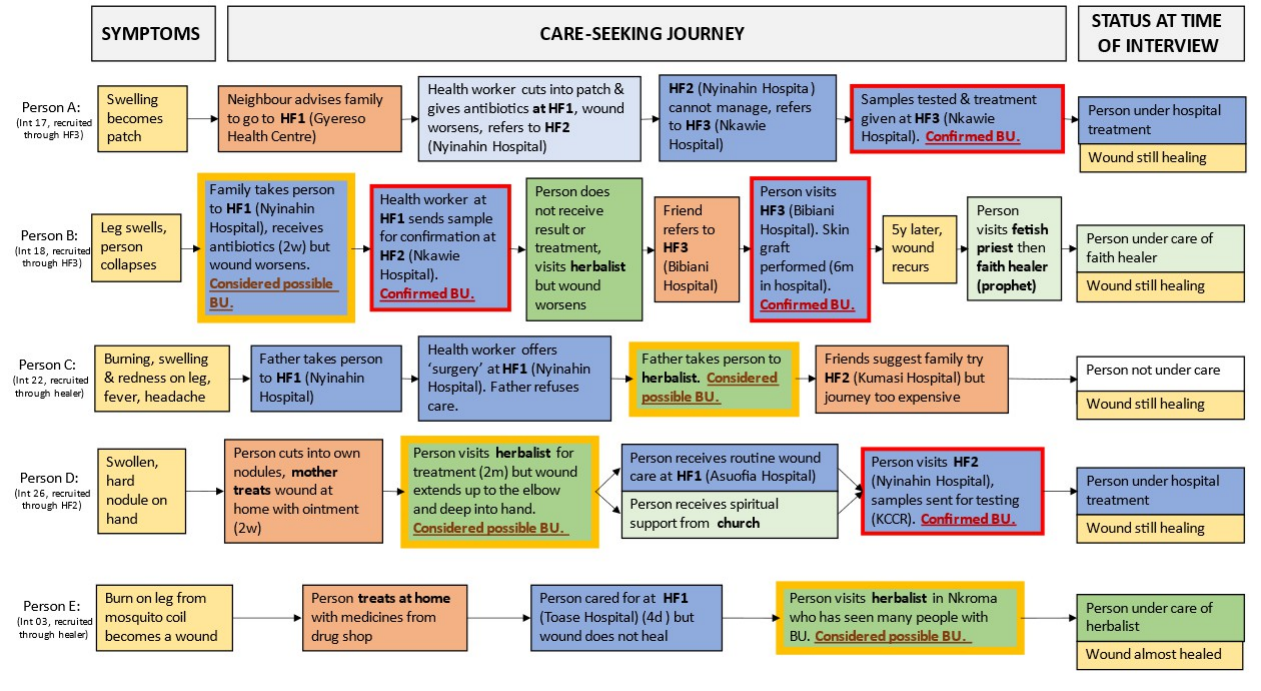
Care-seeking pathways of five people confirmed (red outlines) or presumed (yellow outlines) to have BU. Signs and symptoms which prompted care-seeking and informed diagnostic decision-making are shown in yellow boxes. Care and advice were provided by a range of people including friends and family (orange boxes), primary health centres (light blue), hospitals (dark blue), herbalists (dark green), and faith healers (light green). No CHPS compounds were consulted. Facilities listed in diagram include places people consulted both within (Gyereso, Nyinahin) and outside (Asuofia, Bibiani, Kumasi/KCCR, Nkawie, Toase) Atwima Mponua District. One herbalist was located outside district (Nkroma). In two case studies (C and E), people presumed to have BU had not been tested in a lab by the time of interview. HF: Health facility.

Provisional diagnoses of BU by traditional healers early in peoples’ care-seeking journeys suggested that lay people in the district “know” BU and commonly consider it a cause of skin problems (Fig 3).

#### Experiences of stigma

People with BU described self-exclusion, reduced feelings of self-worth, and concealment of ulcers, which could result from prolonged experiences of social stigma. Descriptions of stigmatising behaviours enacted by others included teasing and gossiping, avoidance, and exclusion:

"… *girls are particular about their skin […] Her friends would not mingle with her if such a person should get the Buruli Ulcer […] That week, she didn’t come to school […] She decided not to come*.”

[Int 1, female teacher]

*“If they have skin NTDs, no one will want to share any item with them in the house because they think you are not clean. […] The wounds are offensive.”*

[Int 35, Health worker]

Some participants indicated that health workers sometimes avoided caring for people presenting with skin problems:

“*To tell the truth*, *some health workers are good while some are not good*. *[…] if they see that you have skin disease*, *they don’t even want to come near you*. *So*, *health workers are like the people in the community*.”

[Participant 8, Men’s FGD, Kotokuom]

Fear of catching BU from wounds was thought to underlie some exclusionary behaviour discussed. One healer described regularly addressing this fear through personal experiences with caregiving:

*"When the people bring their sick person, they do ask me if the disease is infectious and I explain to them that if the liquid from the wound touches another person, they too can get it. But not as if you get it by sitting next to the person. In my dream, I was told that my household should not shun the people who come here with their condition, and until today, none of my family members have suffered from the disease."*

[Int 7, traditional healer]

Stigma-related coping strategies included self-isolation, drinking alcohol, and concealing wounds, including on public transport during care-seeking journeys and in health facility waiting areas. Some family and community members, however, demonstrated supportive attitudes, which were appreciated by those with BU. One described:

“*Those who are close and love you will not shun you*. *Me*, *for instance*, *some people in the neighbourhood regularly check on me*.”

[Participant 1, People with BU FGD]

#### Economic burden of skin NTDs

Skin NTDs were described as having a substantial economic impact on individuals and households. Some affected individuals had to be selective in the type of work they engaged in, avoiding more physical activities or those requiring specific dress-codes, resulting in job losses and reduced attendance at work and school.

“*The only work I think I can do is the one that requires me to sit at a particular place–anything that involves my hands without the use of my legs*, *like cracking cocoa pods open*.”

[Int 2, man with BU wound on his leg]

Affected people described various strategies to cope with the economic burden of having and seeking care for skin NTDs. Some described benefiting from effective risk-pooling arrangements, including the national health insurance scheme (NHIS) and community-level support; however, even where present, these strategies left gaps. Many relied on their families and savings and were sometimes forced to reduce their expenditure on food or other necessities. The economic burden thus extended to other household members, who in some cases lost their livelihood due to caring for an affected person or assumed financial responsibility for them.

### Care-seeking pathways

Skin problems were managed at home and in traditional healer settings, health facilities, and drug shops (Fig 3). Treatment was often said to commence at home and be self-managed with practices including cutting, cleaning, heat application, and use of pharmaceutical creams and plant-based treatments. Most patients used care from both health facilities (mainly hospitals inside and outside the district) and traditional healers.

Some health workers and healers counselled self-management with medicines or dressing materials purchased locally and advised returning for supervised care at specified intervals or in case of deterioration. Affected individuals, caregivers, and practitioners made decisions to stop care or transfer between providers based on perceived response to available treatments:

*“No, we didn’t see any improvement when we sent her to the hospital. We started seeing improvement when the man at the drug store treated her for 4 months. Then, from there, we sent her to the herbalist at Nkroma and he has helped heal her wound completely.”*

[Int 6, caregiver of woman with BU]

Healers were sometimes responsible for provisional skin NTD diagnosis (persons C, D, E in Fig 3), although people under their care did not always subsequently seek confirmation or care at hospitals.

#### Seeking care from traditional healers

Three main types of traditional healers were consulted by affected people: herbalists, who primarily treat ailments with plant medicines and may or may not call on spirits to help heal wounds; fetish priests, who primarily use spiritual interventions invoking local deities or ancestors to cast out spiritual forces thought to cause the condition or prevent its healing; and faith healers, who pray to Christian or Islamic gods for intercession.

Herbalists were often consulted early on and sometimes guided patients on how to manage plural care:

*“All you need at the hospital is injection to ease the pains. After that, the patient should be brought to me to continue the treatment process. […] Even the hospitals do refer people to me to manage. […] And truly, when they come and I commence my work, the disease ceases to spread […though] if the wound is very big, it can take up to one year to completely heal.”*

[Int 7, traditional healer]

Spiritual healers were consulted when individuals experienced lack of response to treatment and feared curses or spiritual work. One man with BU who experienced wound recurrence after extensive time under the care of a herbalist and a hospital for skin grafting, explained:

“*The truth is that when this happens to you*, *[*…*] you might think that it’s juju [black magic / witchcraft] and so when someone recommends a place for you*, *you go without hesitancy*.”

(Participant 4, People with BU FGD).

#### Seeking care at health facilities

Community representatives described children as more likely to present at health facilities because parents wanted to avoid long-term complications, bullying, school absence, and exclusion from activities when at school. Women were considered more likely to present at health facilities because they “care more” about the appearance of their skin, and have more opportunities to talk to health workers, for example, during educational sessions targeting mothers. Others discussed ‘shyness’ leading people to cover their wounds to avoid embarrassment or stigma, which could cause some to avoid health facilities.

In interviews with affected people, however, neither shyness nor cosmetic concerns were strong features of care-seeking decisions. Rather, in one, fear of amputation motivated a woman with BU and her family to attend the hospital she had been referred to outside of the district:

*“It is just a matter of us taking it seriously for it to heal faster, else they might amputate the leg of a nice lady like this. That was what worried me.”*

[*Int 6, Male caregiver of female with BU*]

#### Financial and economic factors influencing care-seeking

Early care-seeking and treatment adherence were highly influenced by cost and ability to pay, especially for BU. Transportation costs were important, as BU patients had to travel frequently to the district hospital. In principle, free BU wound dressings are available through NHIS, but frequent shortages were reported, which compelled individuals to purchase supplies. Care-seeking also resulted in loss of schooling and work.

The anticipated cost of seeking care at hospitals and health centres sometimes contributed to some individuals’ decision to seek care from traditional healers, because payment could be delayed until harvest time when funds were available. Traditional healers were often nearer than health facilities and sometimes made home visits, further reducing transport and opportunity costs.

*“I have a friend who was suffering from such issue. He went to a doctor for treatment. Whenever he goes for treatment there’s an amount of money that he pays. But when he goes to the herbalist, […] sometimes they don’t charge any money. I think that’s why people go to herbalist more than the hospital.”*

[Participant 2, Men’s FGD]

Herbalists sometimes kept personal stocks of dressing materials, razor blades, and cleaning agents for clients who could not afford to bring their own; however, they were often as poor as their clients. One explained:

*“I am doing this work at the expense of my farm work.”*

[*Int 7, traditional healer*]

This same herbalist made clear that, despite the desire to help his community members, he expected those whom he healed to “come back to show appreciation” in the form of monetary or in-kind payments.

### Policy landscape

According to several of our informants, historically in Ghana, when funds become available for skin NTDs, they tend to be invested in screening campaigns and referral hospitals. NTD policy documents in Ghana show a lack of detail on decentralisation that perhaps reflects this lack of direct experience with decentralisation. In the 2021–25 GHS NTD Masterplan, which follows a template provided by WHO, a key focus of strategies for BU, yaws, and leprosy is case search and active surveillance, with activities integrated into plans for mass preventive chemotherapy interventions where possible. The Masterplan also calls for health systems capacity strengthening to achieve skin NTD programme targets at all levels [8]. In practice, however, few specific activities have been included to achieve a policy objective on decentralisation within primary healthcare and no national guidance indicates which skin NTD resources should be available at each level of the health system. Other districts with donor support have adopted models in which services are decentralised to different degrees. For example, informants described that in Agogo, a community in another district in Ashanti Region, ‘core’ BU care happens at the hospital, but the district has a strong network of community-based surveillance volunteers who bring people for fortnightly treatment visits, while wounds are dressed at non-hospital facilities in the interim.

Key actors interviewed at national, regional, and district levels corroborated many of the observations and concerns raised by community members and health workers in Atwima Mponua, particularly the substantial difficulties and financial constraints of accessing skin NTD care. Actors at all levels believed skin NTDs were not prioritised in Ghana, which resulted in inadequate funding and reliance on financial and technical support from donors and non-governmental organisations. They noted that externally funded projects did not cover all districts, and for districts that were covered, supplies were limited. Low and fluctuating numbers of people needing skin NTD care contributed to challenges in coordination of donated drugs at regional level.

Respondents described health worker training and knowledge of skin NTDs as limited, largely because of logistical and financial challenges of delivering training. They expressed a desire to expand learning opportunities for health workers beyond the district capital. They raised concerns that presumed skin NTD cases are often referred to disease control officers at the district hospital, resulting in bottlenecks and missed opportunities to build capacity within non-hospital health facilities and to provide care closer to affected individuals. GHS officials expressed scepticism of the contributions of drug sellers and particularly traditional healers to skin NTD care and concern that they delayed access to government services:

*“We are… trying to orient clients on the availability of treatments because they always associate these conditions with spirits. And the herbalists and spiritualists within the communities could take advantage of it and hold them for ransom for a long period. So, it is [only] when they [patients] get to the [health] centre and they also refer them to the district that we are able to help them.”*

[KII 07, district health official]

The Masterplan states that a potential challenge for integrated programming is “vertical structures and systems [which] may result in staff of NTD [programme]s resisting change” [8]. None of our informants appeared resistant to decentralising skin NTD care; however, national actors displayed markedly different perspectives on resource availability and therefore the feasibility of service decentralisation compared with regional and district colleagues. National actors reported that drugs and treatments were free and diagnostic resources were available, whereas those at the district level reported limited diagnostic capacity, frequent unavailability of essential items in facilities, and challenges obtaining treatments stored at regional facilities. District respondents felt that in practice, testing and treatment referral pathways were unclear and often reliant on informal networks of personal connections between health workers across different facilities.

## Discussion

This study provides valuable insights into the experiences of people affected by skin NTDs in Atwima Mponua, and those providing or responsible for providing skin NTD services in endemic communities in Ghana. Consistent with substantial literature on the importance of formative research in intervention design, this assessment triangulated multiple information sources using several research methods [31, 32]. We found that experiences were shaped by current health service provision, economic factors, and community perceptions of skin NTDs and available treatment options. Overall, widespread awareness of the limited care capacities in health centres and CHPS facilities and the high direct and indirect costs of formal care-seeking contributed to decisions to visit traditional healers and to delay formal care-seeking at hospitals. Affected people therefore faced fragmented care, which resulted in challenges in obtaining a timely formal diagnosis and subsequent care.

We found that beyond the district hospital, there were major gaps in staff training and supply chains, which contributed to delays in diagnosis and may help explain why reporting of surveillance data from peripheral facilities was so limited. Knowledge of skin NTDs was limited among health workers at peripheral health facilities and the lay public. Within communities, knowledge of BU appeared better than for other skin NTDs, which seemed to facilitate some early provisional or presumed diagnoses by lay people and traditional healers. We found moral associations with skin NTDs to be complex and dependent both on popular disease causation discourses about each skin NTD, and also on the characteristics of the person affected and their susceptibility to transmission or to attracting diseases as a supernatural problem. Although we saw no strong evidence that stigma strongly influenced care-seeking behaviour, affected individuals did describe self-exclusionary behaviour from social situations and reported feeling stigmatised by some health workers.

Many of our findings resonate with previous work. For example, our finding that people with skin NTDs faced out-of-pocket payments at health facilities, even though Ghana’s NHIS notionally covers treatment of skin diseases (including skin NTDs), reflects systemic problems with the NHIS; only half of Ghana’s population is enrolled in the NHIS, and even for those with a valid subscription, delayed claim payments, frequent facility stock-outs, and unapproved charges result in out-of-pocket expenditure [33]. Our findings on illness representations and stigma echo other studies from Ghana, which found yaws to be associated with dirty water and witchcraft [11] and BU to be associated with working, bathing, swimming in or drinking unclean water [12, 34–37]; insect bites [12, 34, 38]; “weak blood” in children [12]; and witchcraft [11, 12, 17, 18, 21, 25, 32]. A minority of people, including health workers, consistently perceive BU as contagious [11, 12, 17, 18, 21, 25, 32] and BU-affected individuals commonly experience stigma [38, 39]. A recent study we conducted with children and adolescents with yaws and BU in Atwima Mponua and a neighbouring district found that school staff contributed to stigmatising practices affecting children with yaws and BU, mainly because of an overinterpreted fear of contagion, and suggested the need for school-based infection control interventions that are sensitive to social context [40]. Our lack of evidence of a predominant role of stigma in shaping care-seeking choices is consistent with the lack of association observed between stigma and care-seeking or treatment delay among BU-affected people across West Africa [38, 41, 42]. Echoing other studies in Ghana and elsewhere [12, 43, 44], individuals with BU and leprosy tended to be open to multiple causality and willing to try many treatments. Reasons for delays in care-seeking at health facilities were therefore usually either that facility-based care had proven ineffective in the past and/or was too expensive to access, supporting findings about the importance of economic factors in shaping skin NTD care-seeking [41].

Some of our findings challenge NTD policies, academic literature, and the experiences and opinions of some national stakeholders. National and international policies recommend the delivery of integrated skin NTD control activities and care provision at the primary healthcare level [7, 8], but we found a lack of appropriate resources to support such integration. Programmes remain largely concerned with single diseases. There has been no restructuring to develop frameworks which might promote integration or decentralisation and possibly optimise limited resources. GHS actors wanted to avoid legitimising the role of traditional healers and drug sellers in a future intervention, despite the Ministry of Health’s institutional support of a traditional medicine council tasked “to change numerous misconceptions about TAM (traditional and alternative medicine)” within the GHS [45] and findings from other skin NTD programmes that involving traditional healers in whole-of-community educational interventions can be productive and even vital for programmatic success [13].

Although some of our empirical findings may be specific to Atwima Mponua, many are broadly generalizable. Some findings pertain to all of Ghana, including, for example, problems with the NHIS [46]; concerns of GHS authorities about formal engagement with traditional healers [47]; and the disconnect between perceptions at the national, regional, and district level of the health service’s level of provision for skin NTDs. Other findings, while conceivably specific to our study district, are likely to be broadly reflective of contexts elsewhere in Ghana; for example, other districts in Ghana which similarly lack specific support for skin NTD care from non-governmental organisations are likely to experience similar gaps in health worker knowledge, medicines, and supplies outside of the district hospital [48, 49]. Other districts without robust skin NTD services and low levels of case reporting may also find populations who have lived with skin NTDs and possess knowledge of symptoms and disease progression. Other NTD literature [50] has shown that while practical, experiential knowledge of disease symptoms is important, ’navigational knowledge’ of how to access care from health facilities is an essential component that complements and ultimately enables successful care-seeking and reporting, and which seemed missing in Atwima Mponua.

Our study has some limitations. We conducted interviews and FGDs with a broad range of relevant stakeholders but the number of people, including children, directly affected by skin NTDs interviewed was small, reflecting the small numbers of cases known to disease control officers and key health workers. These small numbers prevented exploration of factors such as gender and age, which may influence the experiences of sub-groups of affected individuals. Our analysis largely focused on experiences of BU because we could not locate more people affected by leprosy and yaws. Leprosy and yaws may result in substantially different experiences of diagnosis, treatment, and stigma, in part because of the difference in duration of illness. Opinions on the quality of available care in the health system could have been skewed by respondents not wanting to criticize the work of health workers and traditional healers, who were considered fellow community members and with whom they regularly interacted. Our health facility data may not fully capture the burden of skin NTDs within the district because we did not review health facility data in neighbouring districts, where, according to our qualitative data, some patients seek care. Finally, our study took place during a period when the COVID-19 pandemic had disrupted health services [8]; however, our period of service data review included 15 pre-pandemic months and our findings indicate wider issues within the health system, which predate COVID-19.

Collectively, our findings suggest that no single overriding issue, if targeted by an intervention, would improve the care cascade; rather, improving access to care and reducing the clinical, economic, and psychosocial impact of skin NTDs is likely to require a holistic, complementary set of interventions to address the wide-ranging challenges we identified [30]. Further research is needed to explore the potential role of stigma and gender in care-seeking [9], how demographic characteristics and power relations intersect with illness representations and care-seeking decisions [51, 52], the potential for engagement between traditional medicine practitioners and the formal health service, as well as to quantify the economic burden of skin NTDs, to characterise the epidemiology of skin NTDs, and to examine health facility readiness in greater depth. Nonetheless, our findings provide important insights, which have informed the design of a strategy to support high quality, integrated, and decentralised care for skin NTDs in Atwima Mponua. We report separately on this strategy development [30] and will conduct a multidisciplinary evaluation of its implementation. We encourage others promoting and implementing integrated strategies for skin NTDs to engage in similar formative research in other settings to ensure that strategies are locally appropriate, acceptable, feasible, and ultimately effective.

## Supporting information

S1 Checklist Inclusivity in global research questionnaire. (DOCX)

S2 Checklist (DOCX)
