# Supplementary material for: Improving experiences of neglected tropical diseases of the skin: Mixed methods formative research for development of a complex intervention in Atwima Mponua District, Ghana
Source: PLOS Glob Public Health. 2024 Jun 13;4(6):e0002833. doi: 10.1371/journal.pgph.0002833 (PMC11175470; doi:10.1371/journal.pgph.0002833)
Supplement: S2 Checklist — (DOCX) [file pgph.0002833.s002.docx]

# Online Supplementary File 1.

**Consolidated criteria for reporting qualitative studies (COREQ): 32-item checklist**

Framework from: Tong A, Sainsbury P, Craig J. Consolidated criteria for reporting qualitative research (COREQ): a 32-item checklist for interviews and focus groups. *International Journal for Quality in Health Care*. 2007. Volume 19, Number 6: pp. 349 – 357

For manuscript title: Improving experiences of neglected tropical diseases of the skin: Formative research for development of a complex intervention in Atwima Mponua District, Ghana

| **No. Item** | **Guide questions/description** | **Response** |
| --- | --- | --- |
| **Domain 1: Research team and reﬂexivity** | | |
| *Personal Characteristics* |  |  |
| 1. Interviewer/facilitator | Which author/s conducted the interview or focus group? | DO, EKO, EKA, LO, RDT, EK, IMS, JN with help from research assistants and remote support from a large team from two Ghanaian and one UK institutions. |
| 2. Credentials | What were the researcher’s credentials? E.g. PhD, MD | Interview team consisted of 8 people with a variety of credentials including completed and in-progress PhDs, in-progress Masters, first degrees and research assistants. |
| 3. Occupation | What was their occupation at the time of the study? | University lecturers, students and researchers. |
| 4. Gender | Was the researcher male or female? | Male and female. |
| 5. Experience and training | What experience or training did the researcher have? | All interviewers had research methods and analysis training through post-graduate degree programmes and/or experience from prior global health research projects (2-15 years of practice, each). |
| *Relationship with participants* |  |  |
| 6. Relationship established | Was a relationship established prior to study commencement? | National and district NTD programme managers were known to several of the authors from working a similar area of work in the same country. These managers were involved in discussions to develop the study and later participated in audio-recorded interviews to share in-depth knowledge on the policy context and possible solutions. They also facilitated initial community meetings and recruitment of HCW participants who then helped recruit others. |
| 7. Participant knowledge of the interviewer | What did the participants know about the researcher? e.g. personal goals, reasons for doing the research | General research objectives were clearly explained, as well as the position of the interviewer in it. |
| 8. Interviewer characteristics | What characteristics were reported about the interviewer/facilitator? e.g. Bias, assumptions, reasons and interests in the research topic | The research team conducting interviews and FGDs consisted of academic staff from two Ghanaian research institutions, with remote support from overseas collaborators. None of the field team members originally came from the study district but were all native speakers of the main local language (Twi) and fluent in other languages commonly spoken in the district (Ewe, Ga, Ga-Adangme and Hausa).  Interviewers and FGD moderators were selected so that research activities could always be conducted in a language of choice by the respondents, though in all activities, the language chosen was Twi.  Participants were asked to express their preference for the gender of the interviewer and any request for an interviewer of a specific gender was met.  Interviewers consisted of people from a variety of disciplinary backgrounds, including social scientists, economists, health systems researchers some of whom had prior experience working on skin NTDs inside and outside Ghana, particularly BU. |
| **Domain 2: study design** | | |
| *Theoretical framework* |  |  |
| 9. Methodological orientation and Theory | What methodological orientation was stated to underpin the study? e.g. grounded theory, discourse analysis, ethnography, phenomenology, content analysis | Content analysis and grounded theory, informed by specific social science theories on skin NTD socio-economic burden, stigma and case detection. |
| *Participant selection* |  |  |
| 10. Sampling | How were participants selected? e.g. purposive, convenience, consecutive, snowball | Surveys and HCW interviews: all government health facilities in the district purposively approached. Interviews with people affected by skin NTDs: snowball sampling from HCWs at facilities reporting cases and further snowballing to include household and community caregivers. FGDs with community members: recommendations of participants from HCWs at facilities in most affected areas. FGDs with HCWs and people affected by skin NTDs: interested participants from prior research activities. Key actors in skin NTD policy landscape: Study team compiled a list of 5 people potentially knowledgeable about skin-NTD care provision from various branches and levels of the Ghana Health Service and development partners. All were approached and agreed to participate. |
| 11. Method of approach | How were participants approached? e.g. face-to-face, telephone, mail, email | Face-to-face and telephone. |
| 12. Sample size | How many participants were in the study? | 45 interviews with HCWs, people affected by skin NTDs and community informants. 7 FGDs, with 6-10 participants per group. 8 key actors knowledgeable about policy landscape. |
| 13. Non-participation | How many people refused to participate or dropped out? Reasons? | No one refused or dropped out of the district-level work on care-seeking. Many of the key actors approached for interviews on the policy landscape could not be reached or could not make time for an interview. |
| *Setting* |  |  |
| 14. Setting of data collection | Where was the data collected? e.g. home, clinic, workplace | Health facilities, informants’ places of work and occasionally in affected peoples’ homes. FGD participants were grouped by sex and age-range in order to explore common experiences. |
| 15. Presence of non-participants | Was anyone else present besides the participants and researchers? | HCW surveys and interviews: No. FGDs: No. Patient case studies: Caregivers and other people from the household were sometimes present and invited to contribute information. Policy actors: No. |
| 16. Description of sample | What are the important characteristics of the sample? e.g. demographic data, date | Qualitative data were collected from a purposely wide range of people knowledgeable about skin NTDs (BU, leprosy, yaws and other conditions) in Atwima Mponua district from February 2021 – July 2021, including affected patient populations, endemic communities and HCWs from all government facilities involved in skin care. Key actors knowledgeable about the policy and intervention context were interviewed July 2021-Feb 2022. |
| *Data collection* |  |  |
| 17. Interview guide | Were questions, prompts, guides provided by the authors? Was it pilot tested? | Qualitative interview guides were semi-structured and FGDs followed topic guides. All guides were translated between English and Twi and piloted. Research tools are available on request. |
| 18. Repeat interviews | Were repeat interviews carried out? If yes, how many? | Yes. Most interviews with HCWs, patients and community informants were single, but some interviews with patients involved multiple interactions. Additionally, some informants participated in more than one type of research activity (survey, interview, FGD). |
| 19. Audio/visual recording | Did the research use audio or visual recording to collect the data? | Interviews and FGDs were audio recorded with participant consent and transcribed and translated into English for analysis. |
| 20. Field notes | Were ﬁeld notes made during and/or after the interview or focus group? | Yes. FGDs were facilitated by two researchers, one who guided the discussion with the other documenting field notes. Notes were also made during and after interviews and community meetings. |
| 21. Duration | What was the duration of the interviews or focus group? | 60-90 minutes. |
| 22. Data saturation | Was data saturation discussed? | Yes. |
| 23. Transcripts returned | Were transcripts returned to participants for comment and/or correction? | No. |
| **Domain 3: analysis and ﬁndings** | | |
| *Data analysis* |  |  |
| 24. Number of data coders | How many data coders coded the data? | 7 people from all 3 institutions. |
| 25. Description of the coding tree | Did authors provide a description of the coding tree? | Yes, key themes explored in data collection and analysis are listed. |
| 26. Derivation of themes | Were themes identiﬁed in advance or derived from the data? | Partly identified in advance and partly derived from the data. |
| 27. Software | What software, if applicable, was used to manage the data? | MAXQDA v2020. |
| 28. Participant checking | Did participants provide feedback on the ﬁndings? | Key findings were presented to community representatives and some but not all participants from formal data collection activities in dissemination and co-production workshops. |
| *Reporting* |  |  |
| 29. Quotations presented | Were participant quotations presented to illustrate the themes/ﬁndings? Was each quotation identiﬁed? e.g. participant number | Yes. |
| 30. Data and ﬁndings consistent | Was there consistency between the data presented and the ﬁndings? | Yes. |
| 31. Clarity of major themes | Were major themes clearly presented in the ﬁndings? | Yes. |
| 32. Clarity of minor themes | Is there a description of diverse cases or discussion of minor themes? | No, we focus on the main story and particularly perceptions and experiences of BU for which qualitative data was most robust. Other findings are reported in companion papers. |
